# Patterns and correlates of nutrition knowledge across five countries in the 2018 international food policy study

**DOI:** 10.1186/s12937-023-00844-x

**Published:** 2023-03-16

**Authors:** Jasmin Bhawra, Sharon I. Kirkpatrick, Marissa G. Hall, Lana Vanderlee, Christine M. White, David Hammond

**Affiliations:** 1 School of Occupational and Public Health, Toronto Metropolitan University, Toronto, Canada; 2 School of Public Health Sciences, University of Waterloo, Waterloo, Canada; 3 Department of Health Behavior, Gillings School of Global Public Health, and Lineberger Comprehensive Cancer Center, University of North Carolina, Chapel Hill, USA; 4 École de Nutrition, Centre nutrition, santé et société (Centre NUTRISS) and Institut sur la nutrition et les aliments fonctionnels (INAF), Université Laval, Québec, Canada

**Keywords:** Nutrition knowledge, Functional knowledge test, Food processing knowledge, International

## Abstract

**Background:**

Nutrition knowledge is an important determinant of diet-related behaviour; however, the use of disparate assessment tools creates challenges for comparing nutrition knowledge levels and correlates across studies, geographic contexts, and populations. Using the Food Processing Knowledge (FoodProK) score – a measure of nutrition knowledge based on consumers’ ability to understand and apply the concept of food processing in a functional task – nutrition knowledge levels and associated correlates were assessed in five countries.

**Methods:**

Adults, aged ≥18 years, were recruited through the Nielsen Consumer Insights Global Panel in Australia (*n* = 3997), Canada (*n* = 4170), Mexico (*n* = 4044), the United Kingdom (UK) (*n* = 5363), and the United States (US) (*n* = 4527). Respondents completed web-based surveys in November–December 2018. Functional nutrition knowledge was measured using the FoodProK score. Linear regression models examined associations between FoodProK score and sociodemographic, dietary behaviours, and knowledge-related characteristics.

**Results:**

FoodProK scores (maximum, 8 points) were highest in Canada (mean: 5.1) and Australia (5.0), followed by the UK (4.8), Mexico (4.7), and the US (4.6). Health literacy and self-rated nutrition knowledge were positively associated with FoodProK scores (*p* < .001). FoodProK scores were higher among those who reported vegetarian/other dietary practices (*p* < .001); made efforts to consume less sodium, trans fats, or sugars (*p* < .001); ≥60 years (*p* = 0.002), female (*p* < .001), and ‘majority’ ethnic group respondents in their respective countries (*p* < .001).

**Conclusions:**

This study found differences in consumers’ ability to distinguish levels of food processing for common foods, with somewhat lower levels of nutrition knowledge in countries with the highest intake of highly processed foods. Nutrition knowledge differences based on consumer characteristics highlight the need for accessible policy interventions that support uptake of healthy eating efforts across populations to avoid exacerbating nutrition-related disparities. Tools such as the FoodProK can be used to evaluate the impact of policies targeting nutrition knowledge across contexts.

## Introduction

Nutrition knowledge, which includes knowledge of concepts such as dietary guidelines and sources of various nutrients [1], is an important determinant of diet-related behaviour [2]. In particular, nutrition knowledge can influence consumers’ ability to identify healthy foods and manage diet-related chronic diseases [3–5]. Nutrition knowledge is influenced by a myriad of factors, including sociodemographic characteristics and socioeconomic status. Research has shown that consumers who are older, female, and have higher income and education perform better on assessments of nutrition knowledge in cross-sectional studies [6–10]. Moreover, nutrition information may be more accessible to consumers with higher literacy, thereby increasing nutrition knowledge [11, 12]. Other behavioral factors more directly connected to nutrition knowledge also warrant exploration, as research has shown that individuals with specific dietary goals or practices may seek out nutrition information to a greater extent than those without diet-related goals [13, 14]. Motivation to change diet-related outcomes, including weight status and management of conditions such as type 2 diabetes, could potentially drive knowledge-seeking behavior [5, 13, 14].

Consumers obtain nutrition knowledge from numerous sources, such as national nutrition policies, dietary guidelines, and food cultures that might influence uptake of or exposure to nutrition information [15–20]. A variety of tools have been used to measure nutrition knowledge across countries [21], with most studies using unique tools tailored to specific study populations [1, 2, 8, 9]. The use of disparate tools creates challenges for comparing nutrition knowledge levels and corresponding determinants across studies, geographic contexts, and populations [21, 22]. This is also a barrier to conducting between-country studies focused on the role and effectiveness of specific nutrition policies in increasing consumer nutrition knowledge. Overall, very few cross-country studies on nutrition knowledge have been conducted [7–9, 23].

The Food Processing Knowledge (FoodProK) score was developed to measure nutrition knowledge based on consumers’ ability to understand and apply the concept of food processing in a functional task [24]. The focus on processing levels is consistent with increased messaging related to minimizing processed food consumption in dietary guidelines [15–19]. Given that processing is not specific to a given population or context, this measure can serve as an indicator of consumer nutrition knowledge that can be used across studies [24], lending to the interpretation of cross-country research in this area.

To this end, the current study sought to compare nutrition knowledge levels based on the FoodProK among adults in five countries: Australia, Canada, Mexico, the United Kingdom (UK) and the United States (US). In particular, this study aimed to expand our understanding of the correlates of functional nutrition knowledge to include not only sociodemographic and socioeconomic characteristics, but also body mass index (BMI), and dietary behaviors that potentially influence interest in nutrition information. Correlations between FoodProK scores and self-reported nutrition knowledge and health literacy were also examined to assess how the FoodProK performed in comparison with these measures across countries, as they may be used as proxies for nutrition knowledge.

## Methods

### Study design and participants

This study used cross-sectional data from the 2018 wave of the International Food Policy Study (IFPS) [25]. Respondents aged ≥18 years were recruited through Nielsen Consumer Insights Global Panel and their partners’ panels and completed web-based surveys in November–December 2018. To be eligible for this study, participants had to be ≥18 years, reside in the target country, and be able to independently complete the survey using a laptop, desktop computer, or tablet. Consent was provided electronically prior to survey completion. The study was reviewed by and received ethics clearance through a University of Waterloo Research Ethics Committee (ORE# 30829). A full description of the study methodology can be found in the IFPS Technical Report [25].

Respondents with missing data for covariates of interest, including ethnicity (*n* = 296), income adequacy (*n* = 182), education (*n* = 69), food shopping role (*n* = 29), dietary efforts (*n* = 122), FoodProK score (*n* = 17), health literacy (*n* = 29), and self-reported nutrition knowledge (*n* = 153) were excluded from analyses. Prior to exclusion, additional analyses were conducted to verify that respondents with missing data for these variables were not different for any demographic outcomes in this study, or with respect to FoodProK scores compared with the rest of the sample (data not shown). The final analytic sample was 22,102.

### Measures

#### Food processing knowledge score

The FoodProK is a functional test of nutrition knowledge based on level of processing [24]. Respondents viewed and rated images of three food products within each of four categories: fruits (apple, apple juice, apple sauce), meat (chicken breast, deli chicken slices, chicken nuggets), dairy (1% milk, cheese block, processed cheese slices), and grains (oats, cereal, cereal bar). Products in each category were selected based on availability in multiple international contexts, and to represent varied levels of processing according to the NOVA food classification system [26]. Each category included a food in Group 1 (“un/minimally processed”/“whole food”), Group 3 (“processed”), and Group 4 (“ultra-processed”) of the NOVA system (Table 1). NOVA Group 2 foods were not included because they are processed culinary ingredients extracted from whole foods (i.e., oils, flours, sugars) [26]. Branding on food packages was removed and generic product names were used to minimize the potential for bias based on brand familiarity.

**Table 1:**
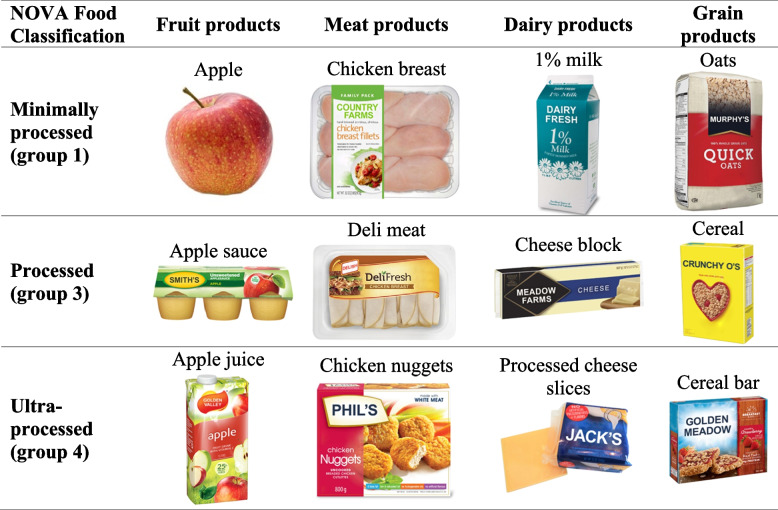
Food Products Included in the Food Processing Knowledge Score Based on NOVA Food Groups

The 12 product images and corresponding Nutrition Facts tables (NFts) and ingredients lists were displayed one at a time, in random order. For each product, respondents were asked, “Overall, how healthy is this food product?” and answered using a scale of 0 to 10, with 0 representing ‘not healthy at all’ to 10 indicating ‘extremely healthy.’

FoodProK scores were calculated based on the concordance of healthiness ratings within each food category with the rankings based on the NOVA classification, with less processed foods representing higher healthiness. Respondents received a full score of 2 if their food product ratings corresponded with the order of NOVA food processing groups (e.g., apple > apple sauce > apple juice). If the respondent ranked 2 of 3 products in a given category in accordance with NOVA (e.g., apple > apple juice > apple sauce), they received a score of 1. Zero was assigned if the respondent’s rankings did not align with those based on NOVA. Respondents’ scores were summed across the four food categories to create the total FoodProK score (ranging 0–8), based on whether they correctly ordered foods according to the NOVA classification for level of processing, with less processed foods representing higher healthiness. Ranging from 0 to 8 [24].

#### Health literacy and nutrition knowledge

Health literacy was measured using an adapted version of the Newest Vital Sign (NVS), which asks respondents six questions regarding an NFt on an ice cream container. The key adaptations to the NVS involved self-administration instead of interviewer-administered questions, as well as changing the NFt design to align with country-specific guidelines (i.e., modifying the font and presentation of nutritional information as required). All NFts were shown in English, with the exception of English and French options in Canada, as well as translating the content to Spanish for the Mexican NFt. The NVS measure assesses respondents’ ability to make mathematical calculations (numeracy), read and apply label information (prose literacy), and understand label information (document literacy) [27]. A score between 0 and 6 was calculated based on the number of correct answers. A score of 0–1 suggests ‘high likelihood (50% or more) of limited literacy;’ a score of 2–3 indicates ‘possibility of limited literacy;’ and a score of 4–6 indicates ‘high likelihood of adequate literacy’ [27]. This measure has been adapted and tested among a variety of age and ethnic groups in different countries including Canada, the US, Australia, and the UK, but has not yet been validated as a self-administered measure [27, 28].

The self-reported nutrition knowledge question was adapted from the Canadian Foundation for Dietetic Research Tracking Nutrition Trends survey [29], and asked, “How would you rate your nutrition knowledge?”, with response options ranging from ‘not at all knowledgeable,’ ‘a little knowledgeable,’ ‘somewhat knowledgeable,’ ‘very knowledgeable,’ to ‘extremely knowledgeable.’ This variable was treated as continuous in analyses (range = 1–5).

#### Consumer dietary behaviours

Individuals engaging in efforts to modify their eating patterns, those practicing vegetarian or other specific dietary patterns, and those with a prominent food shopping role in their households, were hypothesized to be more interested in nutrition, and therefore, also have higher nutrition knowledge [13, 14]. Efforts to modify eating patterns – hereon referred to as ‘dietary efforts’ – were measured by asking, “Have you made an effort to consume more or less of the following in the past year?” Respondents answered, ‘consume less,’ ‘consume more,’ or ‘no effort made’ for each of a list of nutrients and food categories [29]. The current analyses focused on efforts in five categories that have received increasing attention in policies such as dietary guidelines within the five countries: ‘trans fats,’ ‘sugars/added sugars,’ ‘salt/sodium,’ ‘calories,’ and ‘processed foods’ [15–19]. A value of − 1 was assigned for responses to ‘consume less,’ + 1 for responses to ‘consume more,’ and 0 for ‘no effort made’ in the five categories. Five points were added to the sum of the five categories to create a scale ranging from 0 to 10, with 0 representing ‘consume less’ responses to all categories, 10 representing ‘consume more’ responses to all categories, and the range between reflecting all other response combinations. Respondents indicated whether they followed vegetarian and/or religious dietary practices by selecting one or more of the following options: ‘vegetarian,’ ‘vegan,’ ‘pescatarian,’ ‘following a religious practice for eating (please specify),’ or ‘none of the above.’ This variable was recoded as binary (no specific dietary practices = 0; one or more dietary practices = 1) [29]. Food shopping role was captured using an adapted version of an item from the United States Department of Agriculture Eating and Health survey [30], “Do you do most of the food shopping in your household?”, with response options ‘Yes,’ ‘no,’ or ‘share equally with other(s).’

#### Sociodemographic variables and body mass index

Differences in nutrition knowledge in relation to sociodemographic and socioeconomic characteristics may contribute to disparities in nutritional health [3, 31, 32]. Sociodemographic covariates of interest included age group (18–29, 30–44, 45–59, and 60+ years), sex (female or male), country (Australia, Canada, Mexico, the UK, the US), education, ethnicity, and income adequacy. Of the 22,824 IFPS respondents, less than 1% (*n* = 113) reported a gender different than their biological sex, which was insufficient for providing robust estimates in modelling. Hence, sex at birth was used as a binary covariate. Education level was categorized in accordance with country-specific criteria (Low/Medium/High). To enable between-country comparisons, ethnic identity was characterized as ‘majority’ in Mexico if they identified themselves as ‘non-Indigenous,’ and ‘majority’ in Australia, Canada, the UK and the US if they identified themselves as ‘White,’ predominantly English-speaking, or non-Indigenous based on country-specific ethnicity questions. Income adequacy was assessed by asking, “Thinking about your total monthly income, how difficult or easy is it for you to make ends meet?” (Very difficult/Difficult/Neither easy nor difficult/Easy/Very easy) [33].

Categorization of BMI followed World Health Organization criteria [34], with self-reported height and weight used to classify respondents based on BMI < 18.5 kg/m^2^, 18.5 to 24.9 kg/m^2^, 25.0 to 29.9 kg/m^2^, and ≥ 30 kg/m^2^. Response options ‘don’t know’ and ‘refuse to answer’ were provided for all survey questions and recoded as missing. Given the large number of cases with missing height and weight data – including those who selected ‘don’t know’ or ‘refuse to answer’ – a separate category for ‘missing’ was created and retained as a response category for analyses.

### Statistical analysis

Statistical analyses were conducted using SAS Studio (SAS Institute, Cary, NC). Data were weighted with post-stratification sample weights constructed using a raking algorithm with population estimates from respective country-based censuses based on age group, sex at birth, region, ethnicity (except in Canada), and education (except in Mexico) [25]. All reported estimates are weighted.

Descriptive statistics were used to summarize the sample profile, mean, and ranges for FoodProK score by country. A multivariable linear regression model was fitted to examine between-country differences in FoodProK scores. This model included an indicator variable for country, as well as 10 covariates, including the knowledge-related, behavioural, and sociodemographic variables described above. Multiple comparisons were conducted to assess all pairwise contrasts for categorical variables. The Benjamini-Hochberg procedure was applied to decrease the false detection rate following multiple exploratory tests [35]. All statistically significant pairwise contrasts are reported after applying the Benjamini-Hochberg procedure, assuming a false discovery rate of 10%.

Content validity testing of the FoodProK score with subject matter experts indicated two items in the meat category – deli meat slices (processed), and chicken nuggets (ultra-processed) – were too similar to allow differentiation of healthiness [24]. Hence, sensitivity tests were conducted to compare the performance of the original FoodProK score to a modified 7-point score excluding the deli meat product, as well as a 6-point score excluding the meat category entirely. Regression models were tested with all three versions of the FoodProK. Spearman’s rank correlation tests were also run with the original 8-point score and the revised 7- and 6-point scoring to examine potential differences between countries and their association with other knowledge-related variables (self-reported nutrition knowledge, health literacy status).

## Results

### Sample summary

A total of 22,824 respondents completed the IFPS survey. A subsample of 22,102 respondents from Australia (*n* = 3997), Canada (*n* = 4170), Mexico (*n* = 4044), the UK (*n* = 5363), and the US (*n* = 4527) were included in the study after removing respondents with missing data on the covariates of interest. Table 2 presents characteristics of respondents included in the analysis, stratified by country. Each country had approximately equal proportions of male and female respondents. In all countries, the greatest proportion of respondents were from the majority ethnic group and reported their income adequacy as “neither easy nor difficult to make ends meet.” The majority of respondents in all countries reported being the primary food shoppers in their households, did not follow specific dietary practices, and were placed in the “adequate” health literacy category.

**Table 2:** Sample Characteristics (*n* = 22,102), International Food Policy Study, 2018

Characteristic	Australia (***n*** = 3997) % (n)	Canada (***n*** = 4170) % (n)	Mexico (***n*** = 4044) % (n)	United Kingdom (***n*** = 5363) % (n)	United States (***n*** = 4527) % (n)
**Age Group**					
18–29 years	21.2 (845)	19.2 (800)	29.8 (1204)	19.1 (1026)	20.7 (934)
30–44 years	26.5 (1060)	24.7 (1029)	32.3 (1305)	24.4 (1307)	25.2 (1141)
45–59 years	24.7 (988)	25.9 (1078)	28.5 (1155)	26.2 (1407)	25.7 (1165)
60+ years	27.6 (1104)	30.2 (1263)	9.4 (380)	30.3 (1623)	28.4 (1287)
**Sex at Birth**					
Male	49.0 (1959)	49.6 (2069)	47.6 (1925)	48.4 (2609)	48.4 (2192)
Female	51.0 (2038)	50.4 (2101)	52.4 (2119)	51.3 (2754)	51.6 (2336)
**Ethnicity**					
Majority	76.0 (3039)	79.6 (3320)	78.7 (3183)	89.1 (4776)	75.9 (3438)
Minority	24.0 (958)	20.4 (850)	21.3 (861)	10.9 (587)	24.1 (1089)
**Education Level**					
Low	42.0 (1682)	41.3 (1723)	19.5 (789)	48.6 (2605)	58.4 (2645)
Medium	32.6 (1302)	33.7 (1407)	13.2 (535)	23.1 (1240)	9.9 (445)
High	25.4 (1013)	25.0 (1040)	67.3 (2720)	28.3 (1518)	31.7 (1437)
**Income Adequacy**					
Very difficult to make ends meet	8.8 (353)	8.5 (353)	12.1 (490)	6.9 (367)	9.6 (435)
Difficult to make ends meet	19.2 (768)	19.7 (822)	31.8 (1286)	18.4 (985)	20.0 (905)
Neither easy nor difficult to make ends meet	37.6 (1502)	36.8 (1534)	38.7 (1564)	36.4 (1955)	33.9 (1535)
Easy to make ends meet	23.5 (939)	22.4 (935)	13.9 (564)	24.5 (1314)	21.8 (987)
Very easy to make ends meet	10.9 (435)	12.6 (525)	3.5 (141)	13.8 (742)	14.7 (665)
**Body Mass Index**					
< 18.5	3.1 (123)	3.3 (136)	2.1 (85)	3.0 (162)	3.5 (157)
18.5–24.9	35.9 (1437)	33.6 (1400)	39.8 (1608)	34.7 (1861)	30.8 (1395)
25.0–29.9	26.4 (1054)	28.7 (1197)	29.9 (1207)	26.8 (1437)	27.8 (1259)
≥ 30.0	21.1 (842)	24.4 (1019)	15.5 (629)	16.8 (903)	27.3 (1235)
Missing	13.5 (541)	10.0 (418)	12.7 (515)	18.7 (1000)	10.6 (481)
**Food Shopping Role**					
Primary shopper	71.7 (2864)	72.0 (3000)	74.9 (3029)	74.2 (3981)	73.3 (3319)
Not primary shopper	7.1 (284)	6.0 (249)	5.1 (205)	4.7 (253)	6.6 (299)
Shared equally with others	21.2 (849)	22.0 (921)	20.0 (810)	21.1 (1129)	20.1 (909)
**Dietary Practices**					
No specific dietary practices	87.0 (3477)	90.3 (3765)	88.1 (3564)	87.0 (4665)	88.6 (4012)
One or more dietary practices (i.e., vegetarian, vegan, pescatarian, religious practices)	13.0 (520)	9.7 (405)	11.9 (480)	13.0 (698)	11.4 (515)
**Dietary Efforts Score**^a^	2.8 (2.2)	2.6 (2.1)	2.6 (2.3)	3.1 (2.1)	3.0 (2.3)
**Health Literacy**					
High likelihood of limited literacy (score 0–1)	26.7 (1040)	19.4 (810)	30.5 (1234)	31.8 (1707)	25.4 (1150)
Possibility of limited literacy (score 2–3)	24.7 (964)	23.2 (966)	31.2 (1261)	20.5 (1097)	20.2 (913)
Adequate literacy (score 4–6)	48.6 (1897)	57.4 (2394)	38.3 (1549)	47.7 (2559)	54.4 (2464)
**Self-reported Nutrition Knowledge**					
Not at all knowledgeable	5.6 (223)	4.1 (169)	2.8 (114)	9.4 (502)	5.8 (263)
A little knowledgeable	31.5 (1261)	30.1 (1256)	30.4 (1228)	39.4 (2111)	28.8 (1306)
Somewhat knowledgeable	41.4 (1653)	44.4 (1850)	53.0 (2141)	35.7 (1914)	41.2 (1864)
Very knowledgeable	17.4 (696)	18.2 (762)	12.2 (495)	12.6 (674)	18.7 (844)
Extremely knowledgeable	4.1 (164)	3.2 (133)	1.6 (66)	3.0 (161)	5.5 (250)

^a^Mean and standard deviation reported for dietary efforts score

### Comparisons across countries and correlates of FoodProK scores

Within each country, the mean scores across food categories were similar, as demonstrated by the narrow range in scores (Table 3). Australia was an exception as it had the widest mean score range across categories (0.9–1.4), including the lowest dairy score and one of the highest mean scores for the fruit category. Within each food category, mean scores were similar across countries, with dairy scoring lowest across the five countries.

**Table 3:** Food Processing Knowledge Score by Country

	Fruit category mean (SD)	Grain category mean (SD)	Dairy category mean (SD)	Meat category mean (SD)	FoodProK score mean (SD)
**Canada**	1.3 (0.6)	1.3 (0.6)	1.2 (0.6)	1.3 (0.6)	5.1 (1.6)
**Australia**	1.4 (0.6)	1.3 (0.7)	0.9 (0.7)	1.3 (0.7)	5.0 (1.8)
**United Kingdom**	1.2 (0.6)	1.2 (0.7)	1.1 (0.7)	1.3 (0.7)	4.8 (1.9)
**Mexico**	1.4 (0.6)	1.1 (0.6)	1.0 (0.6)	1.3 (0.7)	4.7 (1.6)
**United States**	1.2 (0.7)	1.1 (0.7)	1.0 (0.6)	1.2 (0.7)	4.6 (1.8)
**Five countries combined**	1.3 (0.6)	1.2 (0.7)	1.0 (0.6)	1.3 (0.7)	4.8 (1.7)

The maximum total for each category is 2.0, and 8.0 for the Food Processing Knowledge (FoodProK) score. *SD* Standard deviation

Based on the linear regression analysis (Table 4), those classified as having ‘adequate health literacy’ or the ‘possibility of limited health literacy’ had higher FoodProK scores compared to respondents with a ‘high likelihood of limited literacy’ (β:1.28; CI:1.21, 1.35; *p* < .001; β:0.76; CI:0.68, 0.84; *p* < .001). Self-reported nutrition knowledge was significantly associated with FoodProK score, as respondents who reported they were ‘very knowledgeable’ (β:0.81; CI:0.67, 0.96, *p* < .001), ‘somewhat knowledgeable’ (β:0.75; CI:0.61, 0.88; *p* < .001), and ‘a little knowledgeable’ (β: 0.65; CI: 0.52, 0.79; *p* < .001) scored higher on the FoodProK compared to those who reported that they were ‘not at all knowledgeable.’ Those who reported being ‘a little knowledgeable’ had lower FoodProK scores than those reporting being ‘somewhat knowledgeable (β:-0.09; CI: -0.15, -0.34; *p*=0.002) or ‘very knowledgeable’ (β:-0.16; CI: -0.23, -0.08; *p*<0.001). Respondents who stated they were ‘a little knowledgeable’ had significantly higher FoodProK scores than those who selected ‘extremely knowledgeable’ (β:0.50; CI:0.34, 0.66; *p* < 0.001), and those who reported being ‘extremely knowledgeable’ had significantly lower FoodProK scores than those who reported being ‘somewhat knowledgeable’ (β:-0.59; CI: − 0.75, − 0.44; *p* < 0.001) or ‘very knowledgeable’ about nutrition (β:-0.66; CI: − 0.82, − 0.50; *p* < 0.001).

**Table 4:** Sociodemographic, behavioural, and knowledge-related correlates of the Food Processing Knowledge Score, International Food Policy Study, 2018 (*n* = 22,102)

	Parameter estimate (β)	95% CI	***p***-value
***Country***			
Australia vs. Canada	0.07	−0.01, 0.14	0.08
Australia vs. Mexico	0.22	0.13, 0.30	*0.001
Australia vs. United Kingdom	0.09	0.01, 0.16	*0.02
Australia vs. United States	0.40	0.32, 0.48	* < .001
Canada vs. Mexico	0.15	0.06, 0.23	* < .001
Canada vs. United Kingdom	0.02	−0.05, 0.09	0.61
Canada vs. United States	0.33	0.25, 0.41	* < .001
Mexico vs. United Kingdom	−0.13	−0.21, − 0.05	*0.002
Mexico vs. United States	0.18	0.10, 0.27	* < .001
United Kingdom vs. United States	0.31	0.23, 0.39	* < .001
***Age group***			
30–44 years vs. 60+ years	−0.17	−0.24, − 0.09	* < 0.001
45–59 years vs. 60+ years	− 0.10	−0.17, − 0.04	*0.002
60+ years vs. 18–29 years	0.13	0.04, 0.21	*0.002
***Sex***			
Female vs. Male	0.26	0.21, 0.32	* < .001
***Ethnicity***			
Majority vs. Minority	0.19	0.11, 0.26	* < .001
***Education Level***			
Medium vs. Low	0.02	−0.05, 0.08	0.58
High vs. Medium	0.01	−0.05, 0.07	0.80
High vs. Low	0.03	−0.03, 0.08	0.40
***Income Adequacy***	−0.02	−0.04, 0.00	0.12
***Body Mass Index***			
< 18.5 vs. 18.5–24.9	−0.19	−0.34, − 0.04	*0.01
25.0–29.9 vs. < 18.5	0.18	0.03, 0.34	*0.02
≥ 30.0 vs < 18.5	0.21	0.05, 0.36	*0.008
Missing vs. 18.5–24.9	−0.32	− 0.41, − 0.23	* < .001
Missing vs. 25.0–29.9	−0.33	0.42, − 0.24	* < .001
Missing vs. ≥30.0	−0.31	−0.41, − 0.21	* < .001
***Food Shopping Role***			
Primary shopper vs. Not primary shopper	0.00	−0.12, 0.11	0.93
Primary shopper vs. Share equally with others	−0.06	−0.12, 0.00	0.05
Share equally with others vs. Not primary shopper	0.06	−0.06, 0.18	0.36
***Dietary Practices***			
One or more dietary practices (i.e., vegetarian, vegan, pescatarian, religious practices) vs. No specific dietary practices	−0.31	−0.39, − 0.23	* < .001
***Dietary Efforts Score***	−0.13	− 0.14, − 0.11	* < .001
***Health Literacy***			
Possibility of limited literacy (score 2–3) vs. High likelihood of limited literacy (0–1)	0.76	0.68, 0.84	* < .001
Adequate literacy (score 4–6) vs. Possibility of limited literacy (score 2–3)	0.52	0.46, 0.58	* < .001
Adequate literacy (score 4–6) vs. High likelihood of limited literacy (0–1)	1.28	1.21, 1.35	* < .001
***Self-reported Nutrition Knowledge***			
A little knowledgeable vs. Not at all knowledgeable	0.65	0.52, 0.79	* < .001
A little knowledgeable vs. Somewhat knowledgeable	−0.09	−0.15, − 0.34	*0.002
A little knowledgeable vs. Very knowledgeable	−0.16	−0.23, − 0.08	* < .001
A little knowledgeable vs. Extremely knowledgeable	0.50	0.34, 0.66	* < .001
Somewhat knowledgeable vs. Not at all knowledgeable	0.75	0.61, 0.88	* < .001
Very knowledgeable vs. Not at all knowledgeable	0.81	0.67, 0.96	* < .001
Extremely knowledgeable vs. Somewhat knowledgeable	−0.59	−0.75, −0.44	* < .001
Extremely knowledgeable vs. Very knowledgeable	−0.66	−0.82, − 0.50	* < .001

*CI* Confidence Intervals. *Variables are significant (*p* < 0.05) after post hoc adjustment using Benjamini-Hochberg procedure. Only significant pairwise contrasts are shown for age group, body mass index, and self-reported nutrition knowledge. R^2^ = 0.24

Respondents engaging in one or more specific dietary practices such as vegetarianism had significantly lower FoodProK scores (β:-0.31; CI: − 0.39, − 0.23; *p* < .001) than those with no specific dietary practices. Respondents who reported efforts to consume less sugar, sodium, trans fat, calories, or processed foods had significantly higher FoodProK scores (β: -0.13; CI: − 0.14, − 0.11; *p* < .001) compared to respondents not making efforts to modify their eating patterns in these areas. Food shopping role was not significantly associated with FoodProK score.

The oldest age group (60+ years) scored significantly higher on the FoodProK than the youngest age group (18–29 years) (β: 0.13; CI: 0.04, 0.21; *p* = 0.002). Respondents aged 30–44 years (β: -0.17; CI: − 0.24, − 0.09; *p* < 0.001) and 45–59 years (β: -0.10; CI: − 0.17, − 0.04; *p* = 0.002) had significantly lower FoodProK scores than those in the 60+ years category. Females scored higher on the FoodProK than males (β: 0.26; CI: 0.21, 0.32; *p* < 0.001). Education and income adequacy were not significantly associated with FoodProK score.

Respondents with a BMI < 18.5 or ‘missing’ BMI data had lower FoodProK scores than those with a BMI between 18.5–24.9 (β: -0.19; CI: − 0.34, − 0.04; *p* = 0.01; β: -0.32; CI: − 0.41, − 0.23; *p* < .001). Moreover, respondents with BMIs between 25 and 29.9 (β: 0.18; CI: 0.03, 0.34; *p* = 0.02) or ≥ 30 (β: 0.21; CI: 0.05, 0.36; *p* = 0.008) had significantly higher FoodProK scores than those with BMIs under 18.5, and those with missing BMI data had significantly lower FoodProK scores compared with respondents with BMIs between 25 and 29.9 (β: -0.33; CI: − 0.42, − 0.24; *p* < 0.001) or ≥ 30 (β: -0.31; CI: − 0.41, − 0.21; *p* < 0.001).

As shown in Table 4, respondents from Australia, Canada, Mexico, and the UK scored significantly higher on the FoodProK compared to respondents from the US (β: 0.41; CI: 0.33, 0.49; *p* < .001; β: 0.33; CI: 0.25, 0.41; *p* < .001; β: 0.18; CI: 0.10, 0.27; *p* < .001; β: 0.31; CI: 0.23, 0.39; *p* < .001, respectively). Several other country contrasts were also significant. Respondents in Australia had significantly higher FoodProK scores than those in the UK (β: 0.09; CI: 0.01, 0.16; *p* = 0.02) and Mexico (β: 0.22; CI: 0.13, 0.30; *p* = 0.001). Canadian respondents had significantly higher FoodProK scores than those in Mexico (β: 0.15; CI: 0.06, 0.23; *p* = < 0.001). Respondents in Mexico had significantly lower FoodProK scores than the UK (β: -0.12; CI: − 0.21, − 0.05; *p* = 0.002).

### Sensitivity analyses

Sensitivity analyses indicated that the FoodProK scoring method did not change the pattern of scores across countries or associations between scores and other variables. Irrespective of whether the FoodProK was in the original 8-point format, 7-point format dropping only deli meat, or 6-point format dropping the entire meat category, the same correlates were significant in the regression model, with no meaningful differences in the parameter estimates. Further, the correlations between FoodProK, self-reported nutrition knowledge, and health literacy status were comparable regardless of the scoring approach.

### FoodProK scores and relationships between knowledge-related variables

Health literacy and the FoodProK score were moderately correlated (*r*_s_ = 0.37, *p* < 0.001). There was a very weak, positive correlation between self-reported nutrition knowledge and each of health literacy (*r*_s_ = 0.09, *p* < 0.001) and the FoodProK score (*r*_s_ = 0.09, *p* < 0.001).

## Discussion

The current study is one of the first to examine differences in nutrition knowledge levels across multiple countries. Based on understanding of levels of food processing, adults from Canada and Australia scored highest on the functional nutrition knowledge test, with adults in the US scoring the lowest. Differences across countries are likely due to a range of factors, including national dietary guidelines and nutrition policies that may influence consumers’ access to and uptake of nutrition information based on the reach and effectiveness of these initiatives [36]. Country-specific dietary patterns or food culture may also play a role in nutrition knowledge among populations, particularly informal channels of nutrition education such as family food practices and cultural beliefs which contribute to consumers’ implicit understanding of a food’s nutritive quality/properties [37–39]. This ‘prior’ knowledge may reinforce messaging from national education campaigns, or on the contrary, conflict with cultural beliefs around healthy eating in some populations [40–42]. Countries with the lowest FoodProK scores – Mexico and the US – also have among the highest levels of consumption of ultra-processed foods across countries [43–48]. Lower scores in these countries may reflect lower levels of knowledge or different social norms in populations in which highly processed foods are ubiquitously available and consumed.

Although some differences in nutrition knowledge scores across countries were statistically significant, the magnitude of differences was modest. The large study sample size resulted in high levels of power; thus, even modest differences were statistically significant in some cases. For example, Canada vs. US and UK vs. US had modest, but significantly different FoodProK scores (β: 0.33, *p* < .001 and β:0.31, *p* < .001, respectively) which are difficult to interpret. Modest differences may also reflect similar content in national nutrition guidelines and labelling policies with respect to the NFts that appear on pre-packaged products, which were displayed to respondents as part of the FoodProK [15–19]. Future research should focus on the impact of new national nutrition guidelines on nutrition knowledge, including evaluations of awareness, comprehension, use, and reach of such guidelines documents and associated campaigns.

Overall, cross-country studies of nutrition knowledge to enable comparisons of the current findings are lacking. Grunert et al. (2012) found that adults in the UK had significantly higher nutrition knowledge than respondents from four other European countries [7]. The authors attributed this finding to the “history of health policies and nutrition-related initiatives,” as well as potential cultural differences among UK respondents compared with the other countries (p. 166) [7]. While specific policies are not described by Grunert et al. (2012) [7], the UK was one of the first countries among the six included in the study to adopt dietary guidelines, which may have contributed to consumers’ general nutrition knowledge. We are unaware of any other studies that have examined differences between the five countries included in the current study.

Respondents who reported efforts to modify their eating patterns scored higher on the FoodProK. Individuals with specific diet-related goals likely have a greater interest in nutrition or may rely on labels and other sources of nutrition information more frequently [13, 14]. Moreover, individuals with dietary preferences may possess greater motivation to obtain nutrition information, which may drive them to improve their knowledge to support specific dietary choices [13, 14]. This study did not find an association between food shopping role and nutrition knowledge, which may reflect the fact that such tasks are gendered and based on the social organization of society rather than nutrition knowledge [49, 50].

Sociodemographic differences in knowledge were also observed. Consistent with other literature, functional nutrition knowledge was higher with age and among females [7, 9, 10]. Existing evidence points to behavioural and attitudinal differences between men and women, as well as different age groups, as a possible explanation for these differences. Women and older age groups appear to be more health conscious, and it is hypothesized that increased interest in healthy eating may result in increased nutrition knowledge due to intentional efforts to seek out nutrition information [7, 51]. Moreover, nutrition and food tend to be predominantly “female domains,“ [49, 51] suggesting women may be more likely than men to be exposed to nutrition-related health information, increasing their opportunities to gain knowledge.

The association between ethnicity and nutrition knowledge has not been extensively studied. This study found that the ‘majority’ ethnic group in each country had significantly higher FoodProK scores when controlling for other covariates. Some studies have used other measures of ethnicity such as citizenship status, showing lower nutrition knowledge levels among immigrant populations [23, 52]. This may be explained, in part, by acculturation, as immigrants in varying stages of assimilation may have different exposure to national dietary guidelines. The amount and type of cultural exposures, among other aspects of immigrant or ‘minority’ experiences, could potentially impact knowledge of country-specific guidance on healthy eating [52–54], as well as familiarity with foods in a new cultural context. Additionally, given racism that excludes some individuals from fully participating in economic and other systems, those not identifying as ‘White’ or ‘non-Indigenous’ may have had fewer opportunities to develop and apply nutrition knowledge and related skills, such as label reading [54–56]. Overall, these factors may result in lower capacity to answer the FoodProK questions.

With respect to BMI, there were notably lower FoodProK scores among those with missing BMI data compared to the other categories, and higher FoodProK scores when comparing the highest BMI categories to the lowest < 18.5 group. Generally, the literature is inconclusive with respect to associations between BMI and nutrition knowledge [57, 58]. Furthermore, this study relied on self-reported height and weight. US-based studies have shown that weight tends to be under-reported [59–62], and while it is unlikely that data are missing at random, it is difficult to discern what might underlie the BMI associations observed in this study.

The findings also shed light on different methods of assessing nutrition knowledge. FoodProK scores were positively associated with a measure of health literacy, the NVS, which provides a functional assessment of respondents’ ability to understand and apply numeric and descriptive information contained in NFts. Given the focus of the NVS on a nutrition label, this measure might be considered to assess nutrition literacy [21]. In contrast, a commonly used measure of self-rated nutrition knowledge, in which participants rate their perceived level of knowledge on a scale of 1 to 5, was very weakly associated with health literacy, as well as FoodProK scores. Respondents who rated themselves as ‘extremely knowledgeable’ had lower literacy and FoodProK scores, which suggests that many respondents drastically overestimate their nutrition knowledge. This finding reinforces the need to move beyond single-item measures towards functional tests of nutrition knowledge, such as the FoodProK, in order to capture some of the nuance and complexity of nutrition knowledge.

The strength of this study lies in the large sample size and multi-country design, which enabled comparisons of nutrition knowledge using a functional measure. Several limitations should also be considered. First, the sample was recruited using non-probability sampling, which does not enable the generation of nationally representative population estimates. Moreover, there is potential for social desirability bias given the use of self-reported measures [59, 60]. There are also limitations of the FoodProK score, as content validity testing demonstrated poorer performance in the meat category compared to other categories [24]. Sensitivity tests revealed the FoodProK score performed similarly irrespective of whether 6-, 7- or 8-point scales were used; however, further validity and reliability testing of this measure is required, including examining its ability to accurately capture nutrition knowledge in diverse populations and contexts. Modest differences in knowledge may be related to the FoodProK test’s limited ability to detect differences in nutrition knowledge. The large study sample further enabled detection of statistically significant differences in small parameter estimates, which may not reflect meaningful differences in nutrition knowledge across subgroups in all cases. In addition, self-administration of the NVS has not been validated. While this limitation is consistent across all countries, future studies should examine potential differences in self vs. interviewer-administered versions.

## Conclusions

In sum, the current study suggests some differences in consumers’ ability to distinguish levels of food processing for common foods, with somewhat lower levels of nutrition knowledge in countries with the highest intake of highly processed foods. Differences in nutrition knowledge based on consumer characteristics highlight the need for policies that are accessible to those with lower literacy and education. Consumers who tend to have higher nutrition knowledge, including females, higher education groups, and those with specific dietary goals, performed better on the FoodProK score. This pattern of findings suggests the need for novel methods to support uptake of nutrition education efforts across populations, with attention to ameliorating existing disparities. Tools such as the FoodProK can be used to evaluate the impact of policies and interventions targeting nutrition knowledge across contexts, advancing the evidence in this area.

## Data Availability

The datasets analysed during the current study are available from the corresponding author on reasonable request.

## Abbreviations

BMI: Body mass index
CI: Confidence Interval
FoodProK: Food Processing Knowledge
IFPS: International Food Policy Study
NFt: Nutrition Facts table
NVS: Newest Vital Sign
UK: United Kingdom
US: United States
